# From Pituitary Stem Cell Differentiation to Regenerative Medicine

**DOI:** 10.3389/fendo.2020.614999

**Published:** 2021-01-19

**Authors:** Maria Andrea Camilletti, Julian Martinez Mayer, Sebastian A. Vishnopolska, Maria Ines Perez-Millan

**Affiliations:** Departamento de Fisiología, Biología Molecular y Celular, Facultad de Ciencias Exactas y Naturales, Instituto de Biociencias, Biotecnología y Biología Traslacional (IB3), Universidad de Buenos Aires, Ciudad de Buenos Aires, Argentina

**Keywords:** stem cells, pituitary, iPSCs, colonies, SOX2, differentiation

## Abstract

The anterior pituitary gland is comprised of specialized cell-types that produce and secrete polypeptide hormones in response to hypothalamic input and feedback from target organs. These specialized cells arise during embryonic development, from stem cells that express SOX2 and the pituitary transcription factor PROP1, which is necessary to establish the stem cell pool and promote an epithelial to mesenchymal-like transition, releasing progenitors from the niche. Human and mouse embryonic stem cells can differentiate into all major hormone-producing cell types of the anterior lobe in a highly plastic and dynamic manner. More recently human induced pluripotent stem cells (iPSCs) emerged as a viable alternative due to their plasticity and high proliferative capacity. This mini-review gives an overview of the major advances that have been achieved to develop protocols to generate pituitary hormone-producing cell types from stem cells and how these mechanisms are regulated. We also discuss their application in pituitary diseases, such as pituitary hormone deficiencies.

## Introduction

Compelling evidence during the last decades has demonstrated the existence of stem cells (SCs) within the vertebrate’s pituitary with capacity to divide and self-renew, and later to differentiate into specialized cell types. These stem cells express the transcription factor SOX2 and the pituitary transcription factor PROP1, which is necessary to establish the stem cell pool and promote an epithelial to mesenchymal-like transition, releasing progenitors from the niche (1). Pituitary SCs are essential during development and throughout the postnatal life, being able to differentiate according to physiological demand or in response to damage of the tissue in a highly plastic and dynamic manner (2, 3).

In this present review, a summary of the key findings amidst the localization of pituitary stem cells in different vertebrate model systems is presented featuring the major approaches employed for their discovery. Finally, we will discuss research advances to generate pituitary hormone-producing cell types from progenitor cells and their possible applications in regenerative therapies in hypopituitarism patients.

## Characterization of Pituitary Stem Cells in Vertebrates

One of the main anatomical differences between the mammalian and the teleost pituitary is the hypothalamic innervation. In mammals, a portal system carries the hypothalamic signals to the pituitary parenchyma, whereas in fish, the pituitary cells are directly innervated by the hypothalamus (4).

**Figure 1:**
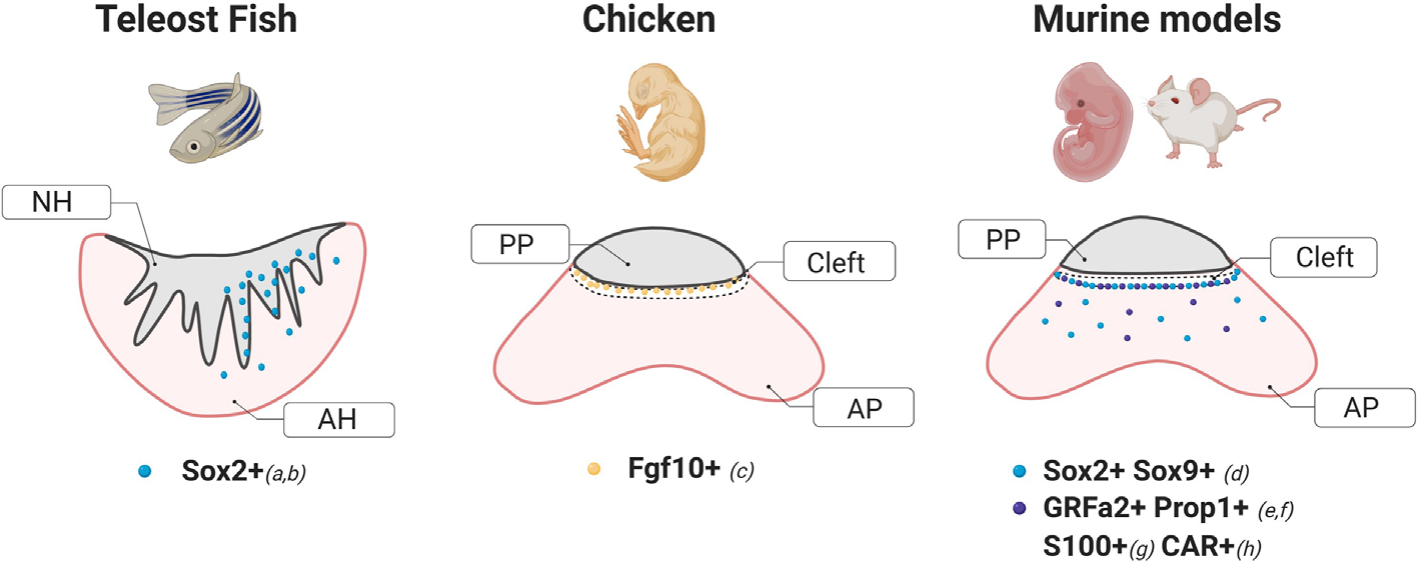
Schematic diagrams of stem cells markers and their localization within a coronal section of the pituitary of different animal models. These cartoons were done based on the following studies: a) Fontaine et al. (6), b) Nakahara et al. (7), c) Fu et al. (8), d) Fauquier et al. (5), e) Garcia-Lavandeira et al. (9), f) Pérez Millán et al. (1), g), Lepore et al. (10), H) Chen et al. (11). NH, Neurohypophysis; AH, Adenohypophysis; AP, Anterior Pituitary; PP, Posterior Pituitary.

Studies in two different species of teleosts, the Japanese rice fish Medaka (*Oryzias latipes*) and the zebrafish (*Danio rerio*) have demonstrated the existence of potentially multipotent SC within the pituitary of these organisms. In Medaka, a significant number of cells expressing the marker Sox2 have been found localized in the dorsal region of the adenohypophysis in close proximity to the *pars nervosa*, with only a few cells dispersed throughout the parenchyma, consistent with its reported location in mammals (5) (**Figure 1**). Specifically, Medaka Sox2+ cells were found in regions of active proliferation evidenced by BrdU incorporation and Proliferating Cell Nuclear Antigen staining (PCNA+) (6). In zebrafish, *in situ* hybridization studies have shown the presence of Sox2+ cells in the anterior pituitary (7) only after 48–72 h postfertilization, suggesting a temporal pattern-expression during development. Although further work is necessary, it is likely that Sox2+ cells found in zebrafish pituitaries were pluripotent stem cells as shown in other tissues including the brain (12) and the retina (13–15).

Several genes found to be essential for pituitary development in mice including the Pou domain transcription factor *Pou1f1*, the protein phosphatase Eya1, and mediators of the Notch signaling were also found in zebrafish, making these models ideal for molecular studies (7, 16–19). However, other factors related to pituitary functions, like Dmrt5, appear to be exclusively found in zebrafish (20).

Compared to other vertebrate model systems, the chicken model (*gallus gallus domesticus*) displays several advantages to study prenatal developmental events, as the relatively large size of its eggs allows the possibility to observe the progress of embryo development in a time-controlled environment. The avian pituitary gland contains several hormone-producing cell types distributed along the caudal and cephalic lobes of the anterior pituitary (AP). These specialized cells arise gradually during development simultaneously with the inflow of hematopoietic cells (21).

Work by Fu et al. in the embryonic chick demonstrated that cells expressing the fibroblast growth factor 10 (Fgf10+) act as both hypothalamic and potential pituitary progenitors. Interestingly, while a group of Fgf10+ progenitors are required for the development of the anterior and mammillary domains, a population of Fgf10+ cells persists in a quiescent state in the tuberal infundibulum which gives rise to the posterior pituitary. In agreement with previous reports in mice (22), hypothalamic factors from the sonic hedgehog (shh) pathway were also found to regulate the differentiation of pituitary cells (8). In addition, transcriptome analysis of the chicken pituitary at 21, 22, and 45 days post-hatching showed a significant enrichment in genes coding for transcription factors known to be critical for pituitary cell development and differentiation such as Pou1f1 and LIM Homeobox proteins 2 (LHX2) and 3 (LHX3) (23).

Particularly in the specification of the Pou1f1 lineage (thyrotrophs, somatotrophs and lactotrophs), microarray and sequencing analysis suggested the involvement of *Ras-dva* in the chicken embryo (24), which is a target of Hesx1, a transcription factor required for proper pituitary development in mice (25). Consistently, further studies by Ellestad et al. showed that corticosterone and Pou1f1 were the main regulators of Ras-dva expression (26).

The identification of pituitary stem cells in non-mammalian animal models is a burgeoning field of research. These cells express pluripotency markers known to have a role in mammalian pituitary development and were found in similar locations as in mammals. However, whether they are multipotent progenitors with the ability to differentiate into hormone-producing cells remains to be defined. In this regard, most convincing evidence comes from studies on mice.

## Adult Pituitary Stem Cells Markers

Several studies have reported mouse pituitary stem cell populations with focus on different markers, mainly: S100, GFRalpha2, Prop1, Sox2, Sox9, Nestin, E-Cadherin and CXCL12/CXCR4. These proteins were proposed as pituitary stem cell markers, and their expression often overlaps within the same cell population.

**Figure 2:**
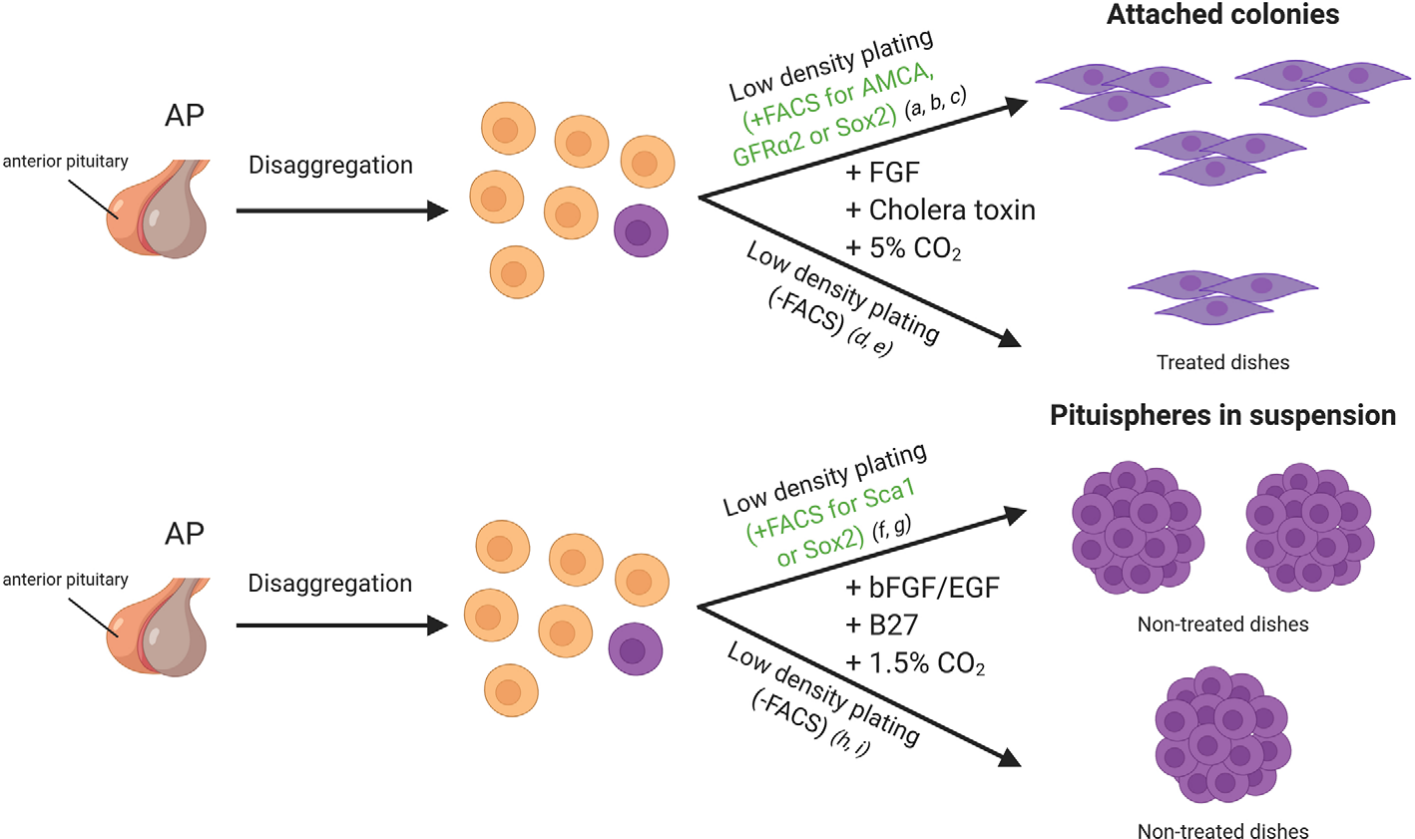
*In vitro* approaches for adult stem cell identification in the mice pituitary gland. Cells of the anterior lobe are dissociated into single cells, and they can be sorted by FACS based on specific characteristics or plated all together. Side population cells display verapamil-sensitive Hoechst dye efflux capacity and/or a stem cell marker such as SOX2, SCA1 and GFRa2. Cells can be seeded in dishes with medium enriched in growth factors (DMEM/Ham’s F12 supplemented with growth factors). References listed correspond to the following articles: a) Lepore et al. (10), b) Andoniadou et al. (27), c) Garcia-Lavandeira et al. (9), d) Gleiberman et al. (28), e) Pérez Millán et al. (1), f) Chen et al. (29), g) Rizzoti et al. (3), h) Chen et al. (30), i) Fauquier et al. (5). FACS, Fluorescence-activated cell sorting.

One of the first approaches used to identify pituitary adult stem cells was based on cell cultures of dispersed mice intermediate and anterior pituitary lobes at low cell density. An experimental observation was that most cells failed to attach to the dish, but some formed small colonies (**Figure 2**). The cells within the colonies resembled folliculo-estellate cells (FS), which express S-100 (10). The presence of S-100+ or GFAP+ cells in the colonies was highly suggestive of FS-cells as a possible source for SC in the pituitary (31). Colony-forming ability of the cells also increased when sorting by intake of *β*-Ala-Lys AMCA, a fluorescent dipeptide derivative that FS-cells incorporate. AMCA+ cells were found scattered across the anterior pituitary, consistent with the reported location of FS-cells, but also importantly in the marginal epithelial cell layer, thus indicating that a different cell-type shared the ability to import AMCA with the FS-cells. Transplantation of these AMCA+ pituitary colony-forming cells into an *in-vivo* system demonstrated a capacity to differentiate into growth hormone (GH) producing cells (32).

Another set of colony-forming SC is the so-called GPS cells (GRFa2/Prop1/Stem) described by Garcia-Lavandeira et al. These cells exhibit a round morphology and form scattered cultures when grown directly on the plates but form more compact colonies when grown on a feeder layer (9). Continued amplification of undifferentiated GPS cells was achieved for several generations, demonstrating their robust self-renewal capacity, a necessary hallmark of stem cells. Interestingly, SOX2 and PROP1 are co-expressed in the pituitary of the mouse embryo and in adult pituitaries (33). PROP1 is a marker of precursors of all hormone-producing cells of the pituitary, and its role in epithelial-to-mesenchymal-like transition (EMT) has been demonstrated (1).

In this context, chemokines were recognized originally for their ability to dictate the migration and activation of bone marrow cells and many cell types, including T cells, dendritic cells, astrocytes, and endothelial cells (34). CXC chemokine ligand 12 (CXCL12, also known as stromal cell-derived factor-1) and its receptor CXCR4 are the first chemokine and receptor that have been shown to be critical for developmental processes, including the pituitary gland (35). Horiguchi et al. found that FS cells secreted CXCL12 and that the CXCL12/CXCR4 axis induces migration, due to chemoattraction, in the anterior pituitary in the formation of the FS cell network. CXCL12/CXCR4 interaction has also been described in human normal anterior pituitary, in both FS and hormone secreting cells (35, 36). They play an important role in GH production and secretion, and in the proliferation of somatotrophs.

Early in the search for pituitary stem cells Chen et al. showed that the primary culture of dispersed AP under neurosphere forming conditions gives rise to *“pituispheres”* (**Figure 2**). The mixing of dispersed APs from wild type and eGFP expressing mice resulted in either eGFP+ or non-fluorescent pituispheres. The absence of heterogeneous eGFP expression implied a clonal origin of the spheres (30). A high percentage of the side population described expressed SOX2 and SOX9, and this subset seemed to be responsible for the formation of the pituispheres described earlier. Strikingly, all pituitary hormones were detected after culturing them with differentiation media, while SOX2 expression faded (29). In some cases, multiple hormones were observed in the same sphere, further supporting the capacity of these cells to give rise to most of the hormonal lineages.

In mice and rats, SOX2+ cells were found distributed in two types of niches, the region lining the AP and the posterior pituitary, also known as the marginal cell layer, and in dense clusters in the parenchyma (5, 37, 38), able to self-renew and give rise to all hormone-producing cell types (3, 27, 39, 40). Further studies in the rat model identified an additional marker associated with the stem cell niche in this region, the Coxsackievirus and adenovirus receptor (CAR) (**Figure 1**). CAR-positive cells showed a similar histological distribution as that observed for SOX2 during development and postnatally, displayed an increased proliferation capacity evidenced by KI67 and expressed epithelial-to-mesenchymal markers like E-cadherin and Vimentin. CAR signals were not detected in the hormonal cell-lineage at any period (embryonic or postnatal), but co-localized with S100β-positive folliculo-estellate cells during adulthood (11).

A pool of SOX2+ cells was also shown to form pituispheres by Fauquier et al. The spheres initially displayed co-expression of SOX2 and E-cadherin, but not S100 or SOX9. However, after a week in culture, both later markers were detectable (5), supporting the hypothesis that FS-like cells observed in early colony formation assays were most likely progenitors and not “true” stem cells. Again, cells from these spheres could be differentiated into all hormone producing cells *in vitro*. Interestingly SOX9+ cells, as well as SOX2+ cells, can form pituispheres, since FACS sorting for these markers from either transgenic mice Sox2^EGFP/+^ or Sox9^iresGFP/iresGFP^ APs produced fluorescent pituispheres (3).

To this day there are multiple important unanswered questions about the pituitary gland development and maintenance during adult life, in particular regarding the participation of embryonic and adult stem cell population in this process. This is due to the limitations of the *in vitro* models. In 2019 Cox et al. established a protocol to generate organoids from pituitary tissue, a great contribution to the *in vitro* experimental models that allows to study the characteristics of pituitary stem cells and the foundations of pituitary organogenesis (41).

These experiments have therefore clearly demonstrated that adult SCs are multipotent, although the essential factors required for multipotency remain unclear due to the heterogeneity of cultured primary pituitary stem cells. Fortunately, the development of human induced pluripotent stem cell-derived pituitary organoids has allowed further dissection of key regulators of stemness, as discussed in the following section.

## Pituitary Stem Cells in Regenerative Medicine

The pluripotent state of the stem cells allows them to differentiate into several other cell types, both *in vivo* and *in vitro*. However, the origin of these stem cells is embryonic, which raises some ethical issues regarding their harvest and use. In this context, Takahashi et al. first developed in 2007 a protocol for reprogramming somatic human cells into an induced pluripotent status by retroviral transduction of Oct3/4, Sox2, c-Myc and Klf4. The iPSCs (induced Pluripotent Stem Cells), obtained from human dermal fibroblasts, resembled human embryonic stem cells in morphology and gene expression patterns, and were able to differentiate into other cell types (42). This first work opened the door for generating patient and disease specific stem cells for research in pathophysiology pathways and personalized treatments. In the following years, other methods using episomal vectors were also established for obtaining iPSC without relying on retroviral/lentiviral vectors (43, 44). This approach enabled the generation of patient specific iPSC without the integration of exogenous DNA, avoiding mutations by DNA integration, therefore bringing us a step closer to the creation of clinical grade iPSCs.

Suga et al. 2011 designed a protocol to differentiate ACTH producing cells from mice stem cells. Briefly, cell aggregations of stem cells were differentiated by incubations with BMP4 into a three-dimensional sphere of hypothalamic tissue cells surrounded by oral ectoderm cells (Pitx1/2+). Upon activation of the Shh pathway using *smoothened* agonist (SAG), some of these oral ectoderm cells began to express Lhx3 and invaginated into the cell aggregate, simulating Rathke’s Pouch development. These Lhx3+ cells later differentiated into all hormone producing cell lineages, and upon addition of Notch inhibitor DAPT, a greater proportion of ACTH producing cells was obtained. Ectopically transplanted cells into the kidney subcapsule of hypophysectomized mice, produced higher ACTH blood levels, spontaneous locomotor activities and longer overall survival in comparison to non-transplanted mice. A similar protocol was then performed for differentiating pituitary cells from human embryonic stem cells (45).

A major caveat in regenerative medicine is that human endocrine cells derived from iPSCs often do not fully recapitulate the functioning levels of normal tissues (46). However, using the previous approach for obtaining ACTH producing cells, Kasai et al. 2020, demonstrated that these cells compared favorably with adult mice adenohypophysis cells, showing that the combined differentiation of pituitary and hypothalamic cells recapitulates the hormonal crosstalk between the two structures (47).

Attempts for obtaining pituitary cells in 2D cultures were also developed. A combination of BMP4, SHH, FGF8, and FGF10 stimuli was used to obtain hormone producing cells in monolayer stem cells in shorter times than 3D cultures; however, this approach relies on cell sorting based on GFP expression coupled to SIX1, challenging the process to adapt this protocol into obtaining pituitary cells from patients’ iPSCs (48, 49).

The use of human cell lines, especially human SC, allows for an expansion in modeling human disease, particularly when there is a lack of an appropriate animal model or when the somatic cell types involved in the disease are difficult to isolate or grow in culture (50). Human-derived pituitary cells differentiated from iPSCs have been used for understanding mechanisms of human pathology such as autoimmune hypopituitarism by anti-Pit-1 antibodies (51).

Mutations in genes like *PROP1, LHX3, LHX4, HESX1, POU1F1, GLI2*, and *OTX2* are found in patients with combined pituitary hormone deficiency. Many genetic variants found in rare diseases are novel, and found in sporadic cases, where it is not possible to show variant segregation along the family, so most of these variants remain to be of uncertain significance in relation to the pathology. Functional testing of these variants is vital then to assess pathogenicity. Matsumoto et al. 2020, used iPSCs derived from patients’ lymphocytes to functionally study a heterozygous variant in *OTX2*. They showed that the patient derived iPSCs were incapable of differentiating into hormone producing cells in contrast to control iPSCs. However, correcting this mutation by CRISPR/Cas-9 methodology modified the capacity of the iPSCs to fully differentiate into pituitary hormonal cells, validating that this variant was the one causing the hormone deficiency (52).

Finally, patient-specific iPSCs could be used for making therapeutic decisions in the context of personalized or precision medicine. Current treatments for hormone deficiencies or pituitary dysfunction mostly rely on recombinant hormone replacement. Though this has significantly reduced patient morbidity and mortality, it still presents some limitations, as drug administration cannot precisely recapitulate the hormonal changes by circadian and/or stress-induced requirements. This is mostly because the healthy pituitary responds to positive and negative regulatory feedback which regulates the amount of secreted hormones, which exogenous hormone replacement cannot address. Regenerative medicine, mostly involves the transplant of differentiated stem cells to correct the genetic or cell defects in patients, thus is a promising future treatment approach. On the other hand, patient derived iPSCs could be used to test pharmacological treatments for correcting the disease or phenotype observed (50).

However, these approaches mentioned before for obtaining pituitary cells in both 2D and 3D cultures still need to be perfected for use broadly in the clinical setting. The main setback with both techniques is the inability to generate the specific hormone producing cells required to combat an individual patient’s pituitary hormone deficiency. Both approaches are fairly good in differentiating ACTH cells, with the other cell types varying in proportion. Gonadotropes are especially difficult to differentiate as they are often found in low frequency. In 2D culture, different proportions of BMP2 and FGF8 added to the medium can promote the change in proportion of pituitary cells. High BMP2 and low FGF8 promotes LH and FSH cells; equal concentration of both BMP2 and FGF8 has a greater proportion of GH and TSH producing cells, while high FGF8 and low BMP2 stimulate POMC+ cells (49). However, the exact *in vitro* conditions to produce a specific pituitary hormone expressing cell are still underdeveloped. We need a higher insight into the molecular mechanisms that regulate pituitary development. These development-in-a-dish models have already helped us understand some of the factors involved in the process of human pituitary development, so they are sure to help us move closer into that objective.

## Conclusion and Perspectives

In summary, several studies support the existence of multipotent stem cells in the adult pituitary from fish to human. Even if not all these studies arrive to the same conclusions, several features, including Sox2 expression, characterization of the side population nature, and the marginal zone configuration, seem to characterize the phenotype of pituitary stem cells. We believe that further characterization of pituitary stem cells could allow a better understanding of the biological basis of some pituitary pathologies including hypopituitarism and adenoma development. While many obstacles remain to be overcome, we envision that the therapeutic potential of iPSCs will attract further research investments in this area to resolve the challenges and allow the identification of novel targets for disease treatments.

## Author Contributions

All authors planned and wrote the paper. MC, JM, and SV designed the figures. All authors contributed to the article and approved the submitted version.

## Funding

PICT 2017-0002 to MP-M. PICT 2018-4239 to MIPM.

## Conflict of Interest

The authors declare that the research was conducted in the absence of any commercial or financial relationships that could be construed as a potential conflict of interest.
